# Sociodemographic and psychosocial correlates of self-compassion in adolescents with physical health conditions

**DOI:** 10.1186/s13034-026-01074-9

**Published:** 2026-03-24

**Authors:** Burak Uslu, Robert Busching, Petra Warschburger

**Affiliations:** Department of Psychology, Counseling Psychology, University of Potsdam, Karl-Liebknecht-Str. 24-25, 14476 Potsdam, Germany

**Keywords:** Self-compassion, Compassionate and uncompassionate self-responding, Adolescents, Physical health conditions, Social support, Psychological distress

## Abstract

**Background:**

Adolescents with physical health conditions (PHCs) face the dual demands of both normative developmental and disease-related burdens. Self-compassion (SC) represents a potentially important resource, yet little is known about its correlates. This study investigated sociodemographic, health-related, and interpersonal predictors of compassionate self-responding (CS) and uncompassionate self-responding (UCS) among adolescents with PHCs.

**Methods:**

In total, 498 adolescents (ages 12–21) with type 1 diabetes, cystic fibrosis, and juvenile idiopathic arthritis completed measures of SC, depression, anxiety, disease severity and duration, parental support, peer group integration, and seeking social support. Hierarchical multiple regressions were conducted separately for CS and UCS. Predictors were entered as follows: (1) sociodemographic variables, (2) health-related factors, and (3) social resources.

**Results:**

CS was primarily explained by social resources, namely parental support, peer integration, and seeking social support. In contrast, UCS was explained by depression, anxiety, and disease severity, alongside lower parental and peer support. The final hierarchical regression models accounted for 27.8% of the variance in CS and 42.9% in UCS.

**Conclusions:**

Findings reveal two distinct pathways that should be considered in future practice: Results for UCS emphasize the need for early identification and targeted intervention, whereas strengthening supportive interpersonal contexts seems crucial with respect to CS.

## Background

Adolescence is defined as the period between ages 10 and 19, extending into emerging adulthood [1]. This stage is characterized by identity formation, new social roles, and evolving peer and family relationships [2]. For adolescents living with a physical health condition (PHC), this period is uniquely complicated, as they must navigate normative developmental challenges while managing disease-related demands (e.g., adherence, symptom control) and an uncertain future—a “dual challenge” that can disrupt daily routines as well as peer integration and identity development [3–5]. Successfully navigating this stage is crucial for long-term psychological well-being and requires mobilizing both external resources (e.g., parental and peer support) and internal resources (e.g., adaptive coping skills and psychological strengths). Within this context of risk and resilience, self-compassion (SC), i.e., treating oneself with kindness and understanding during moments of suffering or perceived inadequacy, has emerged as a particularly potent resource [6].

SC comprises three interrelated components, each with a corresponding negative counterpart: self-kindness versus self-judgment, common humanity versus isolation, and mindfulness versus over-identification [7]. These components are often conceptualized as two broader dimensions: compassionate self-responding (CS) and uncompassionate self-responding (UCS) [8, 9]. Meta-analytic evidence points to the potential of higher SC to lower depression, anxiety, and stress while augmenting greater well-being and health-enhancing behaviors, leading also to less rumination, thought suppression, and greater psychological flexibility [10–13].

These benefits are particularly relevant for individuals managing PHCs, where SC can help to reduce self-blame under stress, support treatment adherence, and foster constructive responses to setbacks [14, 15]. Research into various PHCs (e.g., cancer, diabetes, and chronic pain) indicates that higher SC is linked to reduced distress, improved treatment adherence, and more effective self-management [16–18]. Longitudinal findings have also described reduced depressive symptoms [19], highlighting its relevance as a psychological resource for this vulnerable population [20].

Despite the compelling evidence of its benefits, little is known about correlates of SC, particularly in adolescents with PHCs. While information on adolescents’ current SC levels may help to identify those at greater psychosocial risk, it does not explain why some adolescents are more vulnerable than others. To address this question, we need to look at contextual influences—such as demographic, health-related, and social factors—that might hinder or encourage SC. Understanding these influences is critical for developing targeted strategies to foster SC and, consequently, prevent psychosocial issues. Therefore, our primary aim was to examine the associations between these factors and SC in this population.

### Gender and age

The importance of gender and age has been consistently demonstrated. Meta-analytic findings have indicated that, on average, females report lower SC than males across various ages [21]. This pattern was shown to be consistent across 65 countries: on average, men reported higher CS scores and lower UCS scores compared to women [22].

With regard to age, cross-sectional research suggests that SC tends to increase from adolescence to adulthood, likely due to age-related gains in self-acceptance, emotional maturity, and perspective-taking [23]. However, some studies have shown that older adolescent females report lower SC than younger girls and boys [24]. This pattern has been attributed to heightened social comparison and fluctuating self-esteem in adolescence, which may hinder the cultivation of a self-compassionate mindset. Consequently, mid-adolescence may represent a stage of vulnerability to lower SC, especially for females. As already mentioned, PHCs may additionally create a high-risk environment that can undermine positive self-perceptions [25].

### Subjective socioeconomic status (SES)

Socioeconomic status (SES) plays an important role by predicting access to instrumental and psychological resources that foster resilience and adaptive coping strategies [26]. Beyond objective indicators, subjective socioeconomic status also linked to psychological adjustment [27]. Adolescents who perceive themselves as lower on the social ladder often experience unfair treatment and social devaluation, leading to discrimination, chronic stress, and self-criticism [28]. Empirical evidence supports this link: Swami and colleagues [22] found that higher financial security and higher educational attainment were associated with higher CS and lower UCS.

### Depression and anxiety

A substantial body of research has established a strong, inverse relationship between SC and psychological distress [29, 30]. While research often positions SC as a protective factor associated with lower distress, it is equally important to consider the reverse pathway, i.e., how psychological and physical health challenges might influence an individual’s capacity for SC. It was stated that negative self-appraisals in depression and threat-related thoughts in anxiety may erode SC [31].

There is consistent meta-analytic evidence that lower SC is strongly associated with higher anxiety and depression among adults and adolescents [10, 11]. This relationship is particularly pronounced among individuals with PHCs: A recent meta-analysis by Baxter and Sirois [32] reported a large inverse correlation between SC and psychological distress within this specific population. Further research suggests that this association is primarily accounted for by the presence of uncompassionate responses—namely, self-judgment, isolation, and over-identification—rather than merely the absence of self-kindness [33].

Illness-related cognitive and affective states may also restrict SC. The core features of depression, such as negative self-appraisals and rumination, create a cognitive climate fundamentally at odds with self-kindness. This is particularly relevant for individuals with PHCs, for whom self-critical judgment has been identified as a key predictor of depression and stress [14]. These negative cognitive-affective patterns are at odds with the core components of SC. Given the moderate to large negative correlations between SC and symptoms of depression and anxiety among adults with PHCs [20], examining its influence among affected adolescents is particularly important.

### Disease severity and duration

Research on illness-related characteristics is limited, but initial findings highlight the relevance of disease severity and disease duration (age at diagnosis). A large study among adults with type 2 diabetes found that a younger age at diagnosis was significantly associated with lower SC, even after controlling for total illness duration [34]. Similarly, in a study including women with endometriosis, illness severity and longer diagnostic delays were linked to lower SC. A recent meta-analysis found no moderation of the association between SC and lower psychological distress by illness duration [32], suggesting that severe or early-onset illness may hinder the development of SC, while its presence remains a potent psychological resource.

### Social resources (parental support, peer group integration, and seeking social support)

Early interpersonal relationships, especially with parents, are pivotal for developing SC. Attachment theory predicts that secure and supportive early bonds with caregivers foster internalized patterns of warmth and self-kindness, whereas rejecting or overcontrolling parenting may promote self-criticism [31, 35]. Adolescents who recall their parents as warm and accepting report higher SC, whereas those perceiving their parents as rejecting or overcontrolling report lower SC [36]. Consistent with this, Neff and McGehee [37] found supportive family environments as significant predictors of SC.

With increasing age, peer relationships become a primary source of social influence and a critical mirror for adolescents to gauge their self-worth [38]. Positive peer interactions can provide emotional support that protects against distress, whereas rejection or conflict can lead to a poorer self-concept and increased vulnerability to anxiety and depression [39].

Social support is a well-established protective factor in psychological adjustment [40], particularly within the context of a PHC [41]. Seeking support as an active coping strategy may represent a behavioral expression of self-kindness, acknowledging that one’s suffering is valid and deserving of care [42]. For adolescents, actively seeking support is particularly important, as they often face fears of burdening family members or doubts that peers can comprehend their experiences [43]. Although prior research has consistently demonstrated a positive association between perceived or received social support and SC [37, 44], to our knowledge, no studies have directly examined the impact of actively seeking social support on SC.

Within the context of PHCs, these three forms of social resources are particularly salient, as disease-related demands may strain parents’ responsiveness, heighten peer isolation, and hinder adolescents’ capacity to seek help.

The present study addressed three main questions: (RQ1) To what extent are demographic factors (gender, age, SES) linked to levels of SC in adolescents with a PHC? We hypothesized that female gender and older age would be negatively associated, and SES positively associated with SC. (RQ2) After accounting for these demographic factors, to what extent do health-related factors (depression, anxiety, disease severity, and age at diagnosis) explain additional variance in SC? We assumed that higher depression, anxiety, greater disease severity, and longer disease duration would be associated with lower SC. (RQ3) After accounting for demographic and health-related factors, to what extent do social factors (parental support, peer group integration, and seeking social support) explain additional variance in SC? We expected parental support, peer group integration, and seeking social support to be positively associated with SC.

## Methods

### Sample and procedure

The current study was part of a larger project within a multicenter consortium (trial registration: DRKS00025125) called COACH (Chronic Conditions in Adolescents: Implementation and Evaluation of Patient-centered Collaborative Health Care) [45]. Data were collected via online questionnaires between June 2019 and November 2021. The inclusion criteria were age 12–21 years and a diagnosis of cystic fibrosis, type 1 diabetes, or juvenile idiopathic arthritis. The final sample comprised 498 adolescents (one participant identifying as non-binary was excluded from gender-based analyses). Descriptive information on participants’ age, gender, SES, disease severity and duration, and PHC are summarized in Table 1.

**Table 1:** Descriptive statistics for demographics and clinical characteristics

	*N*	*M (SD)*	Range
Age	498	15.43 (2.07)	12–21
Gender	498		
Male	207	41.6%	
Female	290	58.2%	
Non-binary	1	0.2%	
SES	480	6.61 (1.42)	1–10
Disease severity	498	2.54 (0.94)	1–5
Disease duration	497	7.61 (4.49)	0–20 (years)
Diagnosis	498		
Type 1 diabetes	388	77.9%	
Juvenile idiopathic arthritis	82	16.5%	
Cystic fibrosis	28	5.6%	

*N*, Sample size; *M*, Mean; *SD*, Standard deviation; SES, Socioeconomic status assessed by the McArthur Scale; Disease duration, Duration since onset of disease in years

### Measures

#### Self-Compassion Scale-Short Form (SCS-SF)

SC was measured with the German version of the Self-Compassion Scale–Short Form (SCS-SF) [46] The SCS-SF consists of 12 items rated on a 5-point Likert-type scale from 1 “*almost never*” to 5 “*almost always*”. The scale was originally conceptualized as representing a single higher-order construct with six components [6] and later tested with a bifactor structure [47], but recent psychometric research has provided compelling evidence for a two-factor structure [48, 49] distinguishing between the two overarching dimensions CS (self-kindness, common humanity, and mindfulness) and UCS (self-judgment, isolation, and over-identification). This two-factor structure is considered theoretically meaningful, as the dimensions demonstrate distinct predictive utility, with UCS often being a stronger predictor of psychopathology [9, 50]. The SCS-SF has demonstrated strong psychometric properties, showing high reliability and a near-perfect correlation with the original 26-item scale [46, 51]. Total scores for each subscale were calculated by averaging their respective items. In the present sample, the internal consistency reached *α* = 0.69 for the CS subscale and *α* = 0.86 for the UCS subscale.

#### Sociodemographic and disease characteristics

Subjective socioeconomic status (SES) was assessed using the MacArthur Scale adapted for adolescents [52]. This scale asks adolescents to rank the socioeconomic status of their family on a 10-step ladder, starting from the lowest step which indicates having the least number of resources and respect. Subjective disease severity was assessed with a single item rated on a five-point Likert scale from 1 “*not at all true for me*” to 5 “*very true for me*”. Lastly, participants self-reported their age and gender (female, male, non-binary) and their age at which a medical diagnosis was given. Disease duration was then calculated by subtracting the age at diagnosis from the participant’s current age.

#### Social resources

Two subscales from the Questionnaire of Resources in Childhood and Youth (FRKJ 8–16; [53]) were used to assess parental support and peer group integration. The items were rated on a four-point Likert scale ranging from 1 “*never true*” to 4 “*always true*”. In the present sample, both subscales demonstrated excellent internal consistency: parental support (*α* = 0.85) and peer group integration (*α* = 0.93).

#### BSSS—The Berlin Social Support Scale

Support-seeking was measured using the respective subscale from the Berlin Social Support Scale (BSSS; [54]). This scale consists of 5 items rated on a four-point scale from “*strongly disagree*” to “*strongly agree”.* Internal consistency in the current study reached *α* = 0.83.

#### Depression

Depressive symptoms were assessed using the German version [55] of the 9-item Patient Health Questionnaire (PHQ-9; [56]). The PHQ-D has a four-point Likert scale ranging from 1 “*not at all*” to 4 “*nearly every day*”. Higher scores indicate a higher level of depressive symptoms. In the current study, internal consistency exhibited *α* = 0.88.

#### Anxiety

Anxiety symptoms were measured using the 7-item Generalized Anxiety Disorder scale (GAD-7; [57]). The GAD-7 items were rated on a four-point Likert scale ranging from 1 “*not at all*” to 4 “nearly every day”. The internal consistency for the scale in the current study was excellent (*α* = 0.91).

### Data analyses

All statistical analyses were conducted using IBM SPSS Statistics for Macintosh, Version 29.0 [58]. Preliminary analyses addressed data accuracy, missing values, and regression assumptions. Missing data were minimal (< 1% for most variables; highest for SES, 3.6%, *n* = 18). Given this low proportion and the lack of external auxiliary variables required to specify a robust multiple imputation model without inflating variance [59], pairwise deletion was applied. This approach yields unbiased estimates when missingness is below the 5% threshold and is assumed to be missing at random [60]. Bivariate correlations (Pearson’s *r*) were calculated to examine interrelations among all study variables.

To test our hypotheses, two hierarchical multiple regression analyses were conducted with CS and UCS as dependent variable. Predictors were entered hierarchically in three blocks: (1) demographics (gender, age, and SES); (2) health-related variables (depression, anxiety, disease severity, and disease duration); (3) social resources (parental support, peer group integration, and seeking social support). Predictor blocks were entered in a theoretically predefined order. No automated variable selection procedures were used. At each block, changes in the explained variance (ΔR²) were tested, and predictors’ contributions were evaluated by standardized beta coefficient (*β*) and *p*-values.

## Results

### Descriptive statistics and preliminary analyses

Prior to testing the main hypotheses, bivariate correlations were calculated (see Table 2). As expected, CS was positively linked with age, SES, parental support, peer group integration, and seeking social support, whereas UCS demonstrated the opposite pattern, correlating positively with depression, anxiety, and disease severity, and negatively with social resources.

**Table 2:** Bivariate correlations among study variables

Variable	1		2	3	4	5	6	7	8	9	10	11	*M*	*SD*
1. CS		.											3.08	0.65
2. UCS		− 0.330^**^	.										2.68	0.91
3. Gender		− 0.020	− 0.184^**^	.										
4. Age		0.118^**^	0.151^**^	0.093^*^	.								15.43	2.06
5. SES		0.170^**^	− 0.144^**^	− 0.101^*^	− 0.105^*^	.							6.61	1.42
6. Disease Severity		− 0.175^**^	0.323^**^	0.062	0.083	− 0.152^**^	.						2.54	0.94
7. Disease Duration		0.015	− 0.043	0.121^**^	0.291^**^	− 0.047	0.058	.					7.61	4.49
8. Anxiety		− 0.277^**^	0.527^**^	0.214^**^	0.091^*^	− 0.186^**^	0.366^**^	− 0.002	.				4.57	3.67
9. Depression		− 0.326^**^	0.513^**^	0.194^**^	0.096^*^	− 0.247^**^	0.336^**^	0.005	0.759^**^	.			5.41	4.28
10. Parental Support		0.302^**^	− 0.410^**^	− 0.084	− 0.135^**^	0.238^**^	− 0.203^**^	− 0.107^*^	− 0.305^**^	− 0.371^**^	.		3.42	0.68
11. Peer Group Integration		0.321^**^	− 0.416^**^	− 0.024	− 0.009	0.243^**^	− 0.189^**^	0.051	− 0.322^**^	− 0.369^**^	0.301^**^	.	3.34	0.58
12. Seeking Social Support		0.437^**^	− 0.336^**^	0.059	− 0.021	0.221^**^	− 0.133^**^	0.001	− 0.237^**^	− 0.379^**^	0.442^**^	0.497^**^	2.69	0.71

*M*, Mean; *SD*, Standard deviation; CS, Compassionate self-responding; UCS, Uncompassionate self-responding; SES, Subjective socioeconomic status; * *p* < 0.05, ** *p* < 0.01 (two-tailed)

### Hierarchical multiple regression analyses

For CS (see Table 3), demographic factors explained in the first step a small but significant proportion of variance (5.2%), with older age and higher SES predicting higher CS. Adding health-related factors increased explained variance; only depression showed a significant prediction. The full model explained 27.8% of the variance, with parental support, peer integration, and seeking social support as the strongest predictors. SES and depression were no longer significant predictors.

**Table 3:** Hierarchical multiple regression summary for Compassionate Self-Responding (CS)

Model	Unstandardized coefficients		Standardized coefficients	*t*	Sig.	95.0% confidence interval for B	
	*B*	Std. error	Beta			Lower bound	Upper bound
Step 1							
Gender	− 0.017	0.060	− 0.013	− 0.281	0.779	− 0.135	0.102
Age	0.047	0.014	0.150	3.305	0.001**	0.019	0.076
SES	0.087	0.021	0.188	4.146	< 0.001***	0.046	0.128
*R*²= 0.052, Δ*R*² = 0.052, *F* (3,470) = 8.63, *p* < 0.001							
Step 2							
Gender	0.063	0.059	0.047	1.071	0.285	− 0.053	0.178
Age	0.054	0.014	0.171	3.801	< 0.001***	0.026	0.082
SES	0.051	0.021	0.111	2.491	0.013*	0.011	0.091
Disease severity	− 0.039	0.033	− 0.055	− 1.183	0.237	− 0.103	0.026
Disease Duration	− 0.002	0.007	− 0.015	− 0.340	0.734	− 0.015	0.011
Anxiety	− 0.016	0.012	− 0.087	− 1.315	0.189	− 0.040	0.008
Depression	− 0.037	0.010	− 0.237	− 3.593	< 0.001***	− 0.057	− 0.017
*R*²= 0.151, Δ*R*² = 0.099, *F* (4,466) = 13.56, *p* < 0.001							
Step 3							
Gender	− 0.010	0.055	− 0.008	− 0.183	0.854	− 0.119	0.098
Age	0.055	0.013	0.172	4.136	< 0.001***	0.029	0.081
SES	0.019	0.019	0.041	0.971	0.332	− 0.019	0.057
Disease severity	− 0.025	0.030	− 0.035	− 0.971	0.411	− 0.084	0.035
Disease duration	− 0.002	0.006	− 0.014	− 0.347	0.729	− 0.014	0.010
Anxiety	− 0.019	0.011	− 0.105	− 1.696	0.091	− 0.041	0.003
Depression	− 0.008	0.010	− 0.050	− 0.771	0.441	− 0.027	0.012
Parental support	0.092	0.045	0.095	2.041	0.042*	0.003	0.181
Peer group integration	0.107	0.054	0.095	1.972	0.049*	0.000	0.214
Support seeking	0.278	0.047	0.302	5.956	< 0.001***	0.186	0.370
*R*²= 0.278, Δ*R*² = 0.127, *F* (3463) = 27.14, *p* < 0.001							

SES , Subjective socioeconomic status; Bold values indicate statistical significance *(*p* < 0.05), **(*p* < 0.01), ***(*p* < 0.001)

For UCS (see Table 4), demographic variables explained 5.9% of the variance. Male gender predicted lower UCS, while older age and lower SES predicted higher UCS. Health-related factors explained an additional 28.6% of variance, with depression, anxiety, and disease severity all predicting higher UCS. In the final model, parental support and peer integration emerged as significant predictors accounting for an additional 8.45% of variance. The full model explained 42.9% of the variance.

**Table 4:** Hierarchical multiple regression summary for Uncompassionate Self-Responding (UCS)

Model	Unstandardized coefficients		Standardized coefficients	*t*	Sig.	95.0% confidence interval for B	
	*B*	Std. error	Beta			Lower bound	Upper bound
Step 1							
Gender	0.313	0.084	0.168	3.744	< 0.001***	0.084	0.168
Age	0.054	0.020	0.122	2.720	0.007**	0.020	0.122
SES	− 0.071	0.029	− 0.110	− 2.451	0.015*	0.029	− 0.110
*R*²= 0.065, Δ*R*² = 0.065, *F* (3,470) = 10.97, *p* < 0.001							
Step 2							
Gender	0.128	0.072	0.069	1.775	0.076	0.072	0.069
Age	0.042	0.017	0.095	2.413	0.016*	0.017	0.095
SES	0.010	0.025	0.015	0.385	0.700	0.025	0.015
Disease severity	0.151	0.040	0.153	3.760	< 0.001***	0.040	0.153
Disease duration	− 0.002	0.008	− 0.010	− 0.259	0.796	0.008	− 0.010
Anxiety	0.063	0.015	0.249	4.260	< 0.001***	0.015	0.249
Depression	0.058	0.013	0.266	4.594	< 0.001***	0.013	0.266
*R*²= 0.345, Δ*R*² = 0.280, *F* (4,466) = 49.73, *p* < 0.001							
Step 3							
Gender	0.176	0.069	0.094	2.561	0.011*	0.069	0.094
Age	0.038	0.016	0.085	2.286	0.023*	0.016	0.085
SES	0.050	0.024	0.078	2.081	0.038*	0.024	0.078
Disease severity	0.125	0.038	0.127	3.314	0.001**	0.038	0.127
Disease duration	− 0.002	0.008	− 0.011	− 0.295	0.768	0.008	− 0.011
Anxiety	0.059	0.014	0.234	4.239	< 0.001***	0.014	0.234
Depression	0.029	0.012	0.133	2.316	0.021*	0.012	0.133
Parental support	− 0.264	0.056	− 0.195	− 4.703	< 0.001***	0.056	− 0.195
Peer group integration	− 0.266	0.068	− 0.169	− 3.943	< 0.001***	0.068	− 0.169
Support seeking	− 0.090	0.058	− 0.070	− 1.551	0.122	0.058	− 0.070
*R*²= 0.429, Δ*R*² = 0.084, *F* (3,463) = 22.67, *p* < 0.001							

*SES* Subjective socioeconomic status; Bold values indicate statistical significance *(*p* < 0.05), **(*p* < 0.01), ***(*p* < 0.001)

As summarized in Table 3, CS was primarily predicted by social resources, particularly seeking social support; conversely, UCS was mainly predicted by health-related and demographic factors (see Table 4). Notably, the explained variance was considerably higher for UCS (42.9%) than for CS (27.8%), underscoring the stronger role of risk factors in relation to UCS.

## Discussion

The primary goal of the present study was to identify correlates of SC in adolescents with PHCs, distinguishing between compassionate self-responding (CS) and uncompassionate self-responding (UCS). Previous research has mainly focused on bivariate associations or outcomes of SC [10, 23, 61], whereas multivariable analyses testing concurrent predictors remain limited [37]. Research on adolescents with PHCs remains particularly scarce [25, 34]. To address this gap, our study investigated the extent to which demographic, health-related, and social factors are associated with SC in this population, taking their intercorrelations into account.

CS was mainly associated with age and social resources. Older adolescents showed higher CS, and although SES was initially associated with CS, this effect disappeared once social variables were considered. Parental support, peer group integration, and seeking social support emerged as strong positive predictors, with support-seeking standing out as the strongest unique contributor. These findings are in line with prior studies demonstrating that adolescents who recall warm and accepting parental relationships report greater SC [36, 37]. Similarly, longitudinal studies suggest that autonomy-supportive parenting fosters SC over time [62]. According to attachment and social mentality theory [35, 63], parental warmth and responsiveness are essential for cultivating a compassionate inner voice.

Concerning peers, findings revealed that adolescents who are integrated into supportive peer groups report higher CS. This is consistent with developmental theories that highlight peers as primary mirrors of self-worth during adolescence. Furthermore, seeking social support emerged as the strongest unique predictor in line with Lazarus and Folkman’s [64] stress coping framework. This suggests that support-seeking may function as a behavioral expression of self-kindness, reinforcing common humanity through experiential feedback [31]. Adolescents with low support-seeking but elevated distress may therefore represent a particularly vulnerable subgroup.

As hypothesized, depression and anxiety were among the strongest predictors of UCS in the final model, even after accounting for social resources. This is in line with cumulative evidence showing that uncompassionate facets of SC are more strongly correlated with psychological distress than compassionate facets [33]. At a process level, depression is characterized by pervasive negative self-appraisal and ruminative, self-referential processing. These processes map directly onto UCS components (e.g., self-judgment, over-identification) and may be related to a reduced sense of common humanity [14]. In turn, anxiety is marked by heightened threat monitoring and worry. These processes maintain a vigilant, self-critical stance and limit mindful, balanced awareness, thereby reinforcing UCS [63]. It is worth noting that, consistent with our finding that UCS—rather than CS—tracks most closely with the severity of depression and anxiety, a meta-analysis study focusing on PHCs indicates a significant inverse correlation between SC and overall distress [32].

Beyond psychological distress, greater disease severity was found to independently predict higher UCS, extending prior research linking symptom burden and pain to lower SC and greater isolation [25, 65]. PHCs often involve recurrent pain, functional limitations, and uncertainty, all of which activate the threat system and prompt self-critical responding [66, 67]. Our findings suggest that this illness-related threat context uniquely contributes to UCS independent of depression and anxiety. This implies that disease burden represents a distinct correlate of UCS rather than merely a proxy for distress. Notably, although depression, anxiety, and disease severity were all associated with higher UCS, none of these variables showed a significant association with CS. This finding supports the idea that risk-laden processes tend to amplify UCS, whereas supportive social contexts are more important for developing CS. Finally, there is broad evidence of a high occurrence of depression and anxiety among individuals with a PHC [19, 68]. This highlights the clinical significance of our finding that adolescents with elevated distress and greater disease severity are especially vulnerable to UCS. They may therefore benefit from approaches that both reduce threat-driven self-criticism and enhance safety, soothing, and connection [15, 16, 20].

Regarding sociodemographic variables, male gender predicted lower UCS [21, 22], while higher SES—though initially protective—was linked to higher UCS once covariates were considered. One possible explanation is that a higher SES may amplify achievement pressures and social comparison, which may link self-worth to performance [69]. However, the present study did not directly assess such mechanisms, and they should therefore be considered exploratory in nature. Interestingly, Bluth et al. [70] similarly found that adolescents with highly educated fathers reported lower levels of SC. While SES was significantly correlated in our data, all correlations were small to medium (< 0.24), which suggests that this is very likely due to a statistical artifact (e.g., shared variance or suppression effects), although it cannot be excluded. Since individuals low and high on SES differ on many variables it is worthwhile to test whether adding additional covariates yields similar results in order to rule out statistical artifacts.

Notably, lower parental support and weaker peer group integration significantly predicted higher UCS, highlighting that the absence of social resources may exacerbate self-critical tendencies. Taken together, these findings underscore that, while social support promotes CS, its absence may intensify UCS.

Consequently, our findings emphasize two distinct pathways. CS was primarily associated with social resources (parental support, peer group integration, and seeking social support), as resilience-promoting factors. UCS, on the other hand, was associated with psychological factors such as psychological distress and illness severity, functioning as risk-amplifying factors. It is worth noting that the full model explained substantially higher variance in UCS (42.9%) than in CS (27.8%), underscoring the stronger role of risk factors in their association with UCS. This finding supports the theoretical relevance of treating CS and UCS as distinct constructs [9], and points to the need for interventions that strengthen social resources and mitigate psychological distress in order to foster healthier patterns of self-relating. Both regression and correlation results converged, associating CS with social support, whereas UCS aligned with psychological distress and illness burden.

### Limitations and strengths

Some limitations should be considered when interpreting our findings. First, given the cross-sectional design of the study, all findings should be interpreted with caution avoiding any directional or causal interpretations. It is important to acknowledge that bidirectional relationships—particularly between UCS and internalizing symptoms (i.e., depression and anxiety) are likely. Within the self-compassion literature, longitudinal evidence regarding the association between self-compassion and internalizing symptoms remains inconclusive. While some studies suggest a unidirectional path where self-compassion predicts future distress but not vice-versa [71, 72] recent large-scale longitudinal research utilizing random intercept cross-lagged panel models has found evidence for a reciprocal cycle at the within-person level [73]. Future studies should make greater use of prospective designs and analyses, such as cross-lagged panel models to further clarify the dynamic and potentially mutual processes between these variables. Second, the study relied on self-report measures, which may have introduced response bias. Third, the internal consistency of the CS was marginal (α = 0.69), notably lower than that of the UCS subscale. This may have attenuated the strength of the observed associations for CS, potentially leading to more conservative estimates of its predictors compared to UCS. Therefore, the findings regarding the CS subscale should be interpreted with caution, as the actual relationship may differ or be underestimated due to measurement error. Finally, although the overall sample was large, the distribution across different diagnoses was highly unbalanced, with most participants diagnosed with type 1 diabetes. Consequently, our findings may primarily reflect the psychological experiences and self-regulatory demands specific to adolescents managing type 1 diabetes, which could limit the generalizability of the findings across the broader spectrum of chronic PHCs. This is supported by recent meta-analytic evidence suggesting that while self-compassion is a universal protective factor, the magnitude of its association with psychological distress varies depending on the types of PHC [32]. These findings propose that the role of self-compassion may be more or less salient depending on the specific clinical population. Therefore, further research incorporating more balanced clinical samples or condition-specific analyses is warranted to investigate these dynamics across a broader range of PHCs. Despite these limitations, the present study has notable strengths, including a large sample, a clinically relevant yet understudied population, and a simultaneous focus on CS and UCS in relation to multiple predictors.

## Conclusion

In conclusion, the present study demonstrates the distinct correlates of CS and UCS in adolescents with PHCs. UCS was most strongly linked to depression, anxiety, and disease severity, while CS was primarily associated with parental and peer support and seeking social support. These findings underscore the significance of early identification of adolescents at risk of low SC, especially those experiencing elevated psychological distress or greater disease severity. At the same time, they highlight the importance of social factors as protective pathways that might be incorporated in preventive interventions. By integrating risk indicators with resilience-promoting pathways, the study underlines the importance of distinguishing between CS and UCS in both research and practice.

Future research should not only examine whether interventions that strengthen social resources (e.g., support from parents and peers) can foster CS, but also explore approaches aimed at mitigating psychological distress (e.g., emotion regulation training and cognitive-behavioral strategies) to prevent the reinforcement of UCS. Addressing both protective and risk pathways may offer the most effective strategy for fostering SC among adolescents with PHCs.

## Data Availability

Fully anonymized data are available from the corresponding author on reasonable request.

## Abbreviations

SC: Self-compassion
CS: Compassionate-self-responding
UCS: Uncompassionate self-responding
PHC: Physical health conditions
SES: Subjective socioeconomic status
